# The Story of the Insane from Year to Year

**Published:** 1907-01-05

**Authors:** 


### THE STORY OF THE INSANE FROM YEAR TO YEAR.

### Eighteenth Annual Series.

### THE WORCESTER COUNTY ASYLUM.

On the first of January there were in residence 1,195 patients, and by the end of the year the number had risen to 1,199; but this slight increase by no means represents the annual increment of asylum patients, as 65 patients had to be boarded out in other asylums. It was, however, hoped that the new asylum at Barnsley Hall would be ready for occupation at the close of 1905. There were 234 first admissions; 35 readmissions; 117 recoveries; 15 discharged relieved; 50 discharged not improved, and 81 died. The total number under treatment during the year was 1,462, and the average daily number resident was 1,197. The recoveries were at the rate of 46.9 per cent. of the admissions; and the deaths were 6.7 per cent. of the average number resident; the first of these percentages being above the average, and the latter below it. Of the deaths, 19 were caused by cerebral and spinal diseases; 14 by consumption; 8 by other lung diseases, and 10 by colitis. Table X shows that 36 of the admissions owed their insanity to various moral causes, including domestic trouble, adverse circumstances, and religion; and among the physical causes, intemperance in drink accounts for 32; hereditary influence for 83; previous attacks for 69; and 59 were of unknown cause. Excepting colitis, there was no outbreak of infectious disease; but a great many patients suffered from it, and one of the staff was attacked, which is comparatively a rather rare occurrence. As 13.5 per cent. of the deaths were due to colitis, it is not to be wondered at that the Committee are considering the advisableness of building a temporary hospital for the isolation of this disease. We hope the step will be successful as there is no disease commonly met with in asylums which causes so much anxiety to the staff. The Worcester Committee "have been in comunication with the Home Secretary as to the inadvisability of allowing criminal patients to be sent to this asylum, and associating with our other patients," and Dr. Braine-Hartwell gives his opinion "that a county asylum is not in any way the place for criminal patients. Their example, influence and habits have a most demoralising effect on the other patients." Asylum Committees and Medical Superintendents have often made similar statements, and The Hospital has again and again called attention to the injustice of sending such criminals to county asylums; but Home Secretaries are hard to move in such matters, and we much fear the Worcester protest will be barren of results. We are glad to learn that the nurses in this asylum are subject to a three years' course of training, and we should like to have learnt more about the system as regards examination, certificates, extra pay given, etc. The Committee record their appreciation of "the able manner in which the Medical Superintendent, and the other officers continue to discharge their duties." The average cost per head per week was 7s. 8½d. Private patients, whose residences are in Worcestershire, pay 15s. per week; but owing to the crowded state of the wards during the year no patients of this class were admitted.

